# Supplementary figures and images for: Restriction site associated DNA sequencing for tumour mutation burden estimation and mutation signature analysis
Source: Cancer Med. 2023 Nov 17;12(23):21545–60. doi: 10.1002/cam4.6711 (PMC10726921; doi:10.1002/cam4.6711)

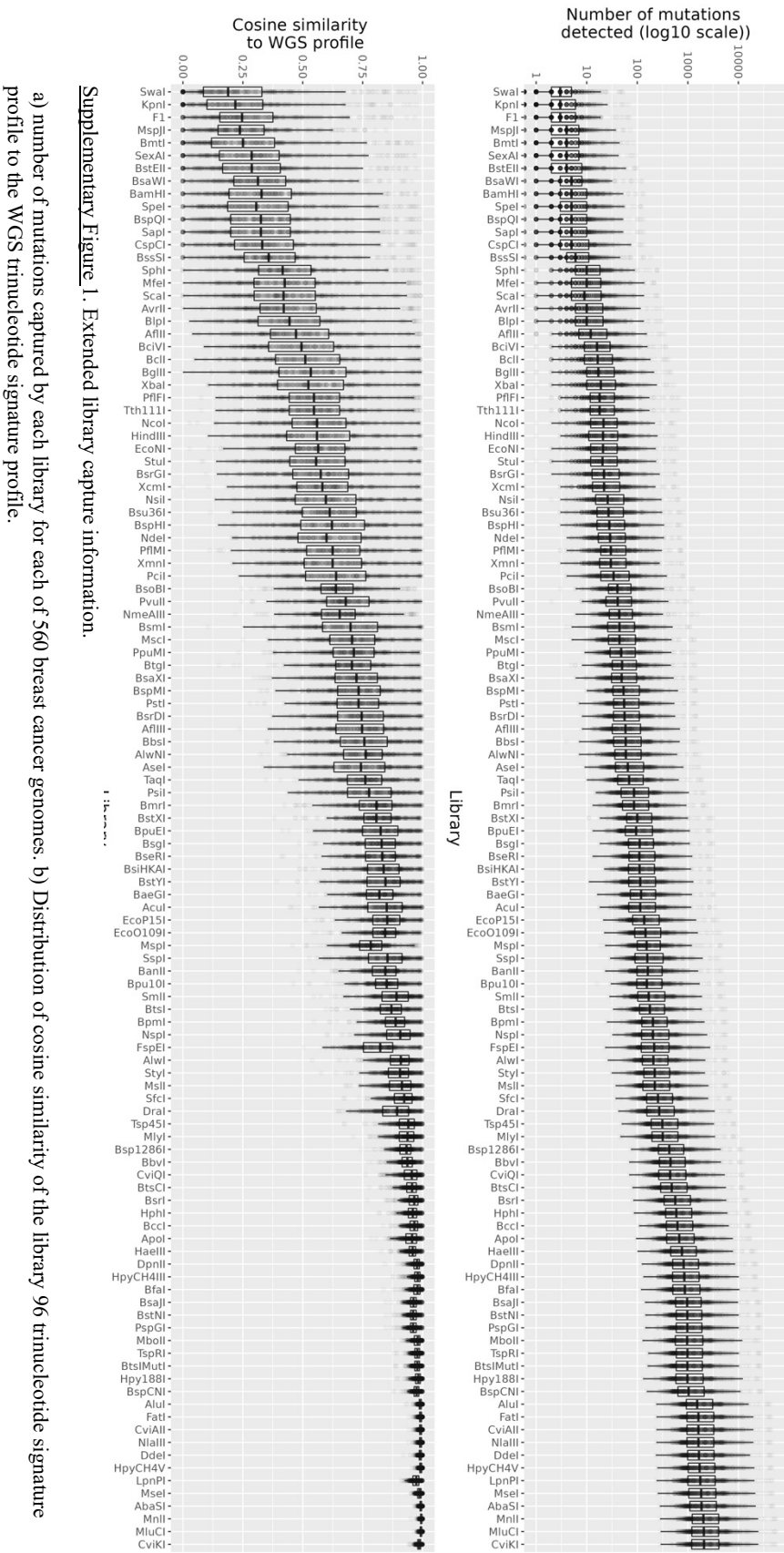

Supplement: Supplementary file 1 — Figure S1 [file CAM4-12-21545-s004.pdf]
