# Supplementary material for: Restriction site associated DNA sequencing for tumour mutation burden estimation and mutation signature analysis
Source: Cancer Med. 2023 Nov 17;12(23):21545–60. doi: 10.1002/cam4.6711 (PMC10726921; doi:10.1002/cam4.6711)
Supplement: Supplementary file 3 — Figure S3 [file CAM4-12-21545-s001.pdf]

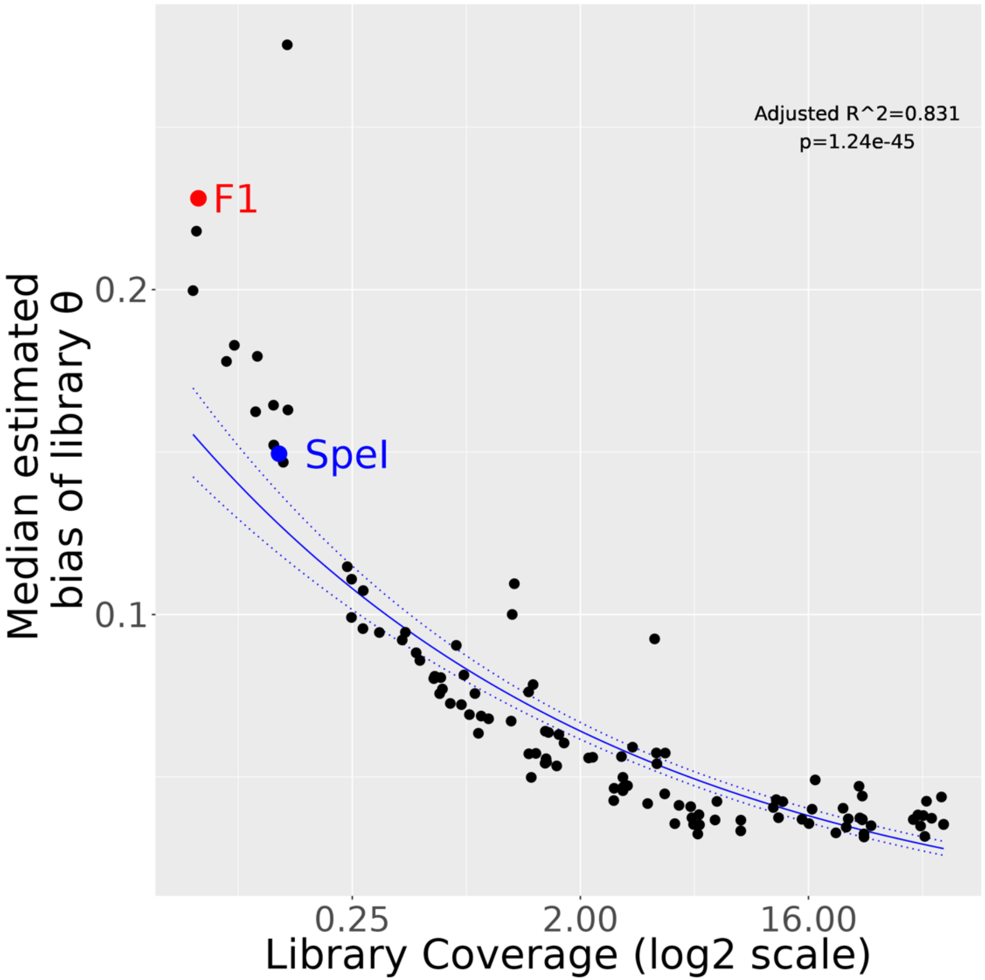

Supplementary Figure 3. Estimated bias θ from Li, Luo (2021) of different libraries by library coverage.

Each point represents a different library. F1 and SpeI libraries are highlighted as red and blue points respectively.
