# Supplementary material for: Restriction site associated DNA sequencing for tumour mutation burden estimation and mutation signature analysis
Source: Cancer Med. 2023 Nov 17;12(23):21545–60. doi: 10.1002/cam4.6711 (PMC10726921; doi:10.1002/cam4.6711)
Supplement: Supplementary file 5 — Figure S5. [file CAM4-12-21545-s003.pdf]

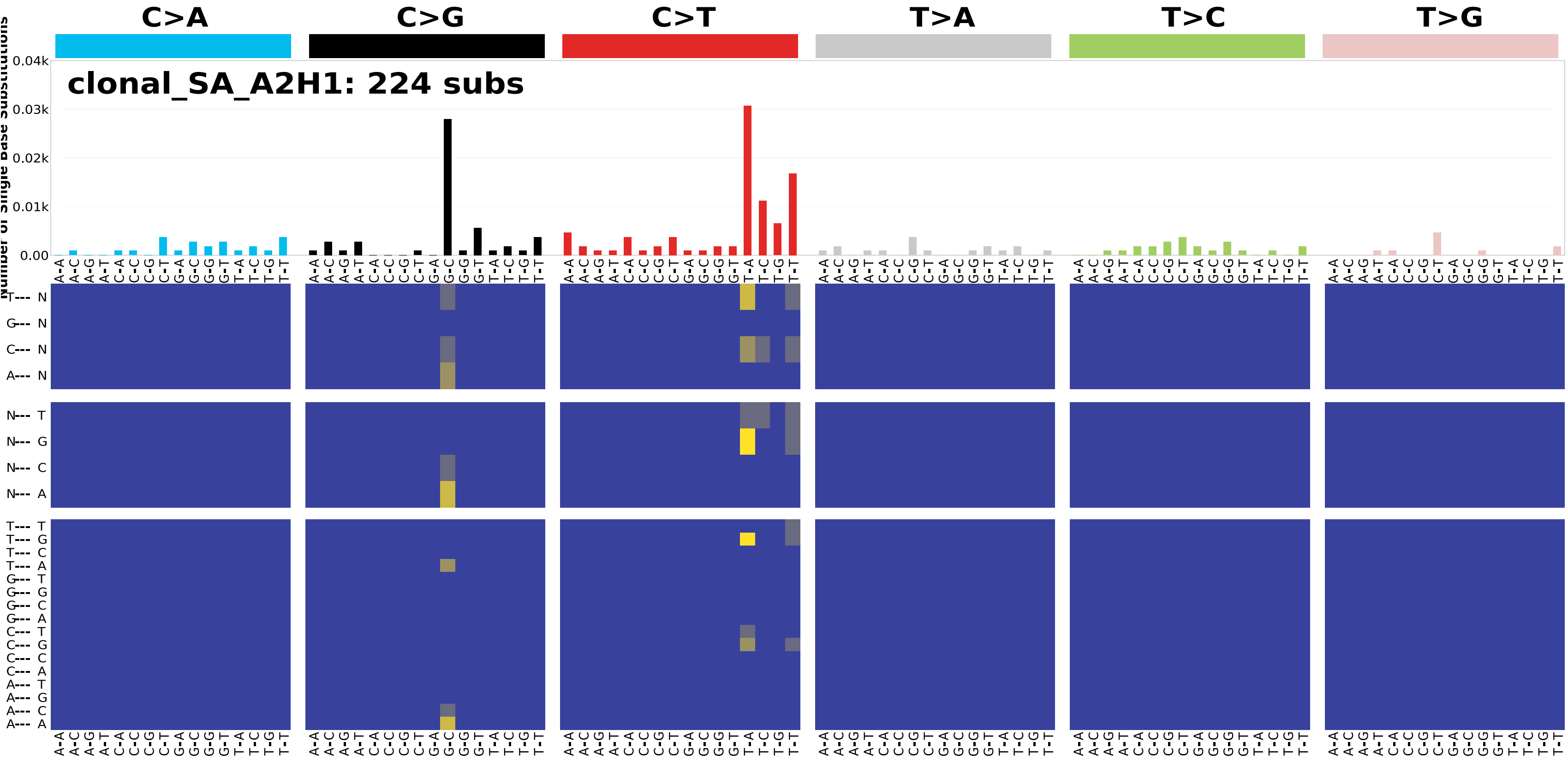

Supplementary Figure 5. Extended 96 mutation profile of the A2H1 clone.

Top: the proportion of mutations attributed to 96 mutation subtypes, coloured by mutation type and ordered by surrounding bases. Bottom: heatmap of relative contributions of mutations in extended contexts: the top heatmap is the extended context considering the bases 5’ of trinucleotide context; the middle heatmap is the extended context considering the bases 3’ of trinucleotide context; and the bottom heatmap is the extended context considering bases 5’ and 3’ of the trinucleotide context.
